# A spectrum of findings on computed tomography in paediatric abdominal and pelvic tumours in a Ghanaian teaching hospital

**DOI:** 10.4314/gmj.v56i4.8

**Published:** 2022-12

**Authors:** Hafisatu Gbadamosi, Yaw B Mensah, Andrea A Appau, Lorner A Renner

**Affiliations:** 1 Department of Radiology, Korle Bu Teaching Hospital, Accra, Ghana; 2 Department of Radiology, University of Ghana Medical School, College of Health Sciences, Accra, Ghana; 3 Paediatric Oncology Unit, Department of Child Health, University of Ghana Medical School, College of Health Sciences, Accra, Ghana

**Keywords:** Paediatric, Abdominal malignancies, Computed Tomography, histology, Ghana

## Abstract

**Objectives:**

To review the Computed Tomography( CT )features of pediatric oncological patients with abdominal and pelvic tumours and correlate these findings with their histopathological diagnosis

**Design:**

This was a retrospective cross-sectional facility-based study.

**Setting:**

This study was conducted in the Pediatric Oncology Unit and Radiology Department of the Korle Bu Teaching Hospital

**Participants:**

Fifty-six pediatric oncology patients with contrast-enhanced abdominal and pelvic CT scans.

**Data Collection:**

The abdominal and pelvic CT scans findings, patient biodata, and histopathology reports of oncology patients over four years were reviewed

**Statistical analysis:**

Simple descriptive statistics using frequency distribution, percentages, means, and standard deviation were used to describe the various variables and presented tables.

**Results:**

The four commonest tumours were nephroblastoma, neuroblastoma, lymphoma, and hepatoblastoma. The mean age at diagnosis was 4.8 years, with a slightly higher male predominance. The majority of the tumours were extremely large at presentation. Overall, the CT – histopathology concordance was 79.2%.

**Conclusion:**

Abdominal and pelvic CT scans play an important role in the diagnostic workup of pediatric malignancies by ensuring early and accurate diagnosis of these tumours.

**Funding:**

None declared

## Introduction

Pediatric malignancies continue to be a leading cause of childhood morbidity globally, with high mortality in low and middle-income countries. Ironically, however, lower estimates of childhood cancer are seen in the middle and lower-income populations, admittedly due to many factors such as – environmental influence, genetic susceptibility, and a major contribution from a failure to diagnose and document these diseases.^1^

Pediatric tumour classifications are generally morphologic rather than site-specific, as seen in adults. They tend to include a more extensive list of differential diagnoses. 2 Multimodality imaging serves as a valuable tool in arriving at the right diagnosis and extent of disease. Modalities such as Ultrasonography (USG), Computed Tomography (CT), Magnetic Resonance Imaging (MRI), and General Radiography (X-ray) are thus usually tailored by a Multidisciplinary team to suit the requirement.^3^

Cross-sectional imaging, including CT and MRI plays a key role during tumour diagnosis and staging vis a vis identifying tumour morphologic features such as margins and density, number of lesions, location, origin, vascular involvement, lymph node involvement and distant metastases. 4 Imaging algorithms and protocols are essential to ensure reproducible practice. The need for more elaborate imaging makes Computed Tomography (CT) and Magnetic Resonance Imaging (MRI) key tools in diagnosing and staging pediatric tumours. 5

CT has been shown to provide excellent anatomic detail, faster acquisition time, large volume coverage, and relatively reduced image degradation by artefact, which are vital in abdominal imaging. CT imaging in childhood is, however, not without risks. The risk of radiation-induced carcinogenesis requires paediatric-specific CT settings, which subject the children to lower radiation dosages than the adult settings. Fortunately, newer multi-detector CT models boast of lower radiation dose techniques/algorithms and shorter scan times. 6 On the other hand, Magnetic Resonance Imaging provides superb soft tissue resolution and is ideal for evaluating the Central Nervous System. However, it is more expensive and less available; it has long image acquisition times with the need for longer periods of specialized sedation than required for CT scans.^7^

The literature on the radiological features of pediatric malignancies in the subregion is scarce, with little information on the CT features and classification of these pediatric abdominal tumours, which this study seeks to document. Being able to define the continuum of imaging findings of these tumours with known histology in our setting will better equip radiologists and clinicians with the appropriate knowledge that will ensure an overall better outcome for these children.

## Methods

A retrospective cross-sectional study was conducted in Ghana's largest hospital, the Korle Bu Teaching Hospital (KBTH) in Accra. It has an established pediatric oncology unit which, in conjunction with the Paediatric Surgery and Radiotherapy departments as well as other specialist units of the hospital, provides comprehensive pediatric oncology care. The hospital manages most pediatric oncology cases in the country and receives referrals from some countries in the West Africa sub-region. The radiology department of KBTH, which provides radiological services, is equipped with two CT scanners - One Toshiba Aquilion, One CT scanner (640 slice), and One Canon Aquilion Start CT scanner (16 slices) as well as one 1.5 T Toshiba Vantage MRI machine.

All available abdomen and pelvic CT studies ( Dicom or hard copy images) of pediatric patients with abdominal and pelvic tumours managed at the Paediatric Oncology Unit of the KBTH between 2016 to 2020 were traced. A total of 56 contrast-enhanced CT scans (Dicom or hard copy images) of patients aged 0 to 17 years were reviewed by two radiologists, each with between 5- and 15 years of working experience, and disagreements were concluded by consensus.

Inclusion criteria were patients aged 17 years and below with complete abdominopelvic CT scan study, without or without a histopathological diagnosis. The exclusion criteria were patients without a complete abdominopelvic CT scan.

### Data Collection and analysis

Data from the review of the patient's records and archived images - including the age of patients, gender of the patient, histopathological diagnosis, abdominopelvic CT scan findings including the location of lesions, tumour characteristics, tumour margin, enhancement pattern, tumour size, effect on vessels, presence, and location of metastases - were compiled on an excel spreadsheet and transferred to Statistical Package for the Social Sciences, SPSS. Simple descriptive statistics using frequency distribution, percentages, means, and standard deviation were used to describe the various variables and presented tables.

### Ethical issue

Ethical clearance was granted by the Institutional Review Board Research and Development Division of the Korle Bu Teaching Hospital - ID NO. KBTH-STC 000165/2020 and permission sought from the Radiology and Paediatric Oncology department. Patient identifiers were anonymised and confidentially maintained throughout the study.

## Results

### Demography

A total of 56 cases were evaluated, of which 27 (48.2 %) were female, and 29 (51.8 %) were males. The most represented age group was 0 - 2 years, with 25 patients (44.6 %), followed by the 5 to 10-year-old group with 13 patients (23.2 %), with the least being the 11 to 17-year group with six patients (10.7 %) as shown in Table 1. The mean age of the study population was 4.8 years, with an SD of 4.1.

**Table 1:** Comparison of histologically diagnosed tumour frequency and age distribution

	AGE-GROUP (YEARS)						

TUMOUR	FREQUENCY N (%)	0–2	3–5	6–10	11–18	Mean	SD
Hepatoblastoma	6 (10.7)	4	2	0	0	6.0	1.54
Leukaemia	2(3.6)	0	0	2	0	6.5	0.71
Lymphoma	9(16.1)	3	1	3	2	6.5	3.5
Malignant germ cell tumour	2 (3.6)	1	1	0	1	8.5	9.19
Mature germ cell tumour	1 (1.8)	0	0	0	1	3.01	3.51
Neuroblastoma	10(17.9)	7	3	0	0	1.83	1.34
Rhabdomyosarcoma	4 (7.1)	2	2	0	0	4.95	2.86
Wilms	17(30.4)	7	4	5	1	4.39	3.63
Undifferentiated sarcoma of the liver	1(1.8)	0	1	0	0	6.00	
Hepatocellular carcinoma	1(1.8)	0	0	1	0	7.00	
Untraced diagnosis	3 (5.4)	1	0	1	1	3	5.20
TOTAL	56(100.2)	25	12	13	6	4.80	4.10

### Computed Tomography findings

The four most common diagnoses were nephroblastoma with 17 patients (30.4%), followed by neuroblastoma with ten patients (17.9%), closely followed by lymphoma with nine patients (16.1 %,) and lastly by hepatoblastoma with six patients (10.7%), shown in Table 1.

### Nephroblastoma (Wilms tumour)

All 17 cases of diagnosed Wilms tumours were intrarenal, with 8 (47.1%) measuring between 10 and 20cm in the widest dimension, 7 (41.1%) measured between 6 and 10cm and only 2 (11.8) cm measured between 3 to 5cm. There were 8 (47.1%) right-sided tumours and 7 (41.7%) left-sided, with two (11.8%) being bilateral.

The majority of the tumours, 9 (52.9%), had lobulated margins, 4 (23.5%) had infiltrating margins with 4(23.5%) showing smooth margins. All of them, 16 (94.1%), demonstrated heterogenous enhancement with cystic areas, possibly due to necrosis or haemorrhage.

**Figure 1:**
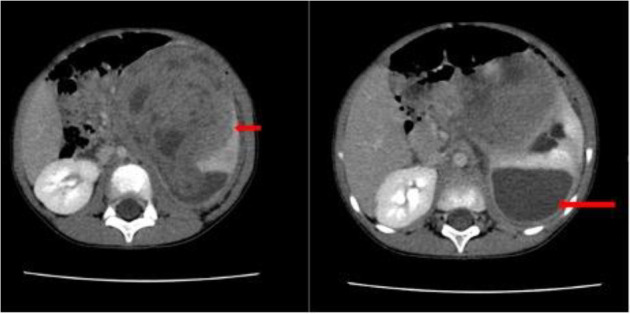
Contrast-enhanced axial abdominal CT scan image of a 6-year-old male child. **a**. Large heterogeneously enhanced left renal mass with a claw sign (short arrow) b. Large posteriorly located subscapular hematoma (long arrow), histologically confirmed as Nephroblastoma.

One case (5.9%) showed a homogenous enhancement pattern. One of the cases (5.9.%) had associated subcapsular hematoma, Figure 1. In 2 (11.8%), there were associated tumoural calcifications on the non-contrast images, which were all curvilinear. Concerning their effect on the vascular structures, 7 (41.2%) of the cases showed displacement of the major vessels, 3(17.6%) demonstrated encasements of the adjacent vessels with 7 (41.1%) of cases not showing any effect on the adjacent vessels. Additionally, tumour thrombus in the renal vein and inferior vena cava was demonstrated in 2 (11.8 %) cases. Four (23.5 %) cases showed regional lymph node involvement, and 5 (29.4%) of the cases showed distant metastasis to the lung and pleura

### Neuroblastoma

The majority of the cases measured 6 cm or more in their widest dimension at presentation, out of which 4 (40%) measured 6 to 10cm, 4 (40%) measured between 10 and 20cm, and 1 (10%) measured more than 20cm. In 1(10%) of cases, the tumour measured between 3 to 5cm, shown in Table 2.

**Table 2:** Widest Dimension of tumour at presentation

	SIZE						

TUMOUR	0–2	3–5	6–10	11–20	>20	NOT APPLICABLE	FREQUENCY n (%)
Hepatoblastoma	0	0	1	5	0	-	6 (10.7)
Leukaemia	1	0		0		1	2(3.6)
Lymphoma	0	1	0	5	2	1	9(16.1)
Malignant germ cell tumour	0	1	1	0	0		2 (3.6)
Mature germ cell tumour	0	0	1	0	0		1(1.8)
Neuroblastoma	0	1	4	4	1		10 (17.9)
Rhabdomyosarcoma	1	0	3	0	0		4 (7.1)
Wilms	0	2	7	8	0		17(30.4)
Undifferentiated sarcoma of the liver	0	0	0	1	0		1(1.8)
Hepatocellular carcinoma	0	0	0	1	0	-	1(1.8)
Untraced histological diagnosis	0	0	1	1	0	1	3(5.4)
TOTAL							56 (100.2)

Six (60%) of these lesions were in the retroperitoneum, 3 (30%) were suprarenal or of adrenal origin, with 1 (10%) was primarily located within the urinary bladder. With respect to the tumour margins, 7 (70%) had lobulated margins, and 3(30%) had infiltrating margins. Majority of the tumours, 9 (90%) showed heterogenous enhancement due to hemorrhagic or necrotic changes, with 1 (10%) demonstrating homogenous enhancement. In addition, 7 (70 %) of non-contrast images showed intratumoural calcification; most of those 6 (8%) were coarse or punctate as opposed to being curvilinear in 1 (14.2%) case. Four (40%) of the cases showed encasement of the abdominal vessels, 3(30%) displacement of the abdominal vessels with 3 (30%) showing no effect on the adjacent vessels. No case of tumour thrombus was noted. Associated regional lymph nodes were seen in 6 (60%) of cases; out of this number, 5 (85%) were regional lymph nodes and 1(16.7%) distant. 1(10 %) case of pleural metastases was noted, while lytic bone and liver metastases were seen in 2 (20%) cases and 1 (10%) case, respectively.

### Lymphoma and Leukemia

In most histologically diagnosed lymphoma, 5 (55%) measured 11 to 20cm in their largest dimension. The smaller lesion size was documented within the renal parenchyma; this was characterized by multiple small renal cortical nodules, measuring less than 2 cm in 1(11.1%) of the lymphoma cases. In a large proportion of 6 (66.6%), they had well-defined margins with poor definitions of their margins in the rest of the cases. An equal proportion of 4 (44.4%) of the tumours showed homogenous and heterogenous enhancement.

In terms of tumour location, 5 (55.6 %) were in the retro-peritoneum, 2(22.2%) presented as diffuse thickening of the small bowel wall, 1 (11.1%) also showed diffuse involvement of the sigmoid colon and rectal wall and another 1(11.1%) showing multiple renal cortical involvements. In 5 (55.5%) cases, the lesions encased the abdominal vessels, while the remaining 4 (44.4 %) did not significantly affect the vessels.

Associated bulky lymph nodes were seen in 5 (55.5%) cases, with no regional lymph nodes in 4 (44.5%). Both lung and splenic metastases were recorded in 1 (11.1%) case. Half of the cases of leukemia 1/2 (50%) presented with hepatosplenomegaly, with the other half presenting with multiple less than 2cm bilateral renal cortical nodules, which can be indistinguishable from lymphoma radiologically. The histological diagnosis of the last suspected case of leukemia could not be traced.

### Hepatic tumours

**Figures 2a, b:**
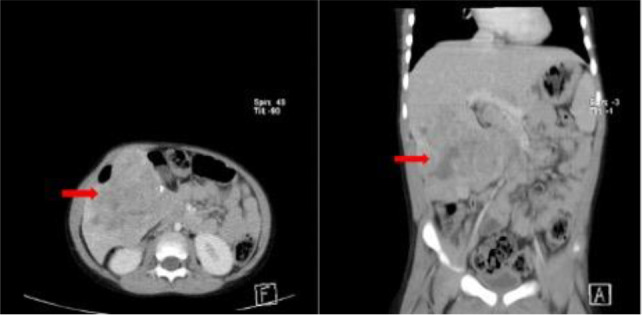
Contrast-enhanced axial abdominal CT scan of a 4 year of male patient showing a lobulated heterogeneously enhancing mass(arrows) in the right lobe of the liver with coarse calcification. Histologically confirmed hepatoblastoma.

The hepatic tumours recorded included hepatoblastoma in 6 (75 %), undifferentiated embryonal carcinoma in 1 (12.5%), and hepatocellular carcinoma in another (12.5%) case. With respect to tumour location within the liver, 3(37.5%) were in the right lobe, 2 (25 %) were in the left lobe, and 3 (37.5%) involved both lobes. Seven (87.5%) of the tumours measured 11 to 20cm, with the majority, 5 (62.5%), being due to hepatoblastoma and the rest being hepatocellular carcinoma (12.5%) and undifferentiated embryonal carcinoma (12.5%). Only one (12.5 %) measured between 6 and 10cm (Figure 2).

All cases demonstrated lobulated margins and heterogeneous contrast enhancement. Four (50%) cases showed coarse and punctate intratumoral calcifications, which were hepatoblastomas. No calcification was noted in the other histological types. Vascular effects, including displacement, encasement, and infiltration of the portal veins, hepatic veins, or IVC, were seen in 5 (62.5%) cases, with no effect in 2 (25%) of cases. To break it down further, 2(22.2%) of the hepatoblastoma were associated with vascular displacement, with another (22.2%) associated with encasement of the vessels. 1(11.1%) of hepatoblastomas demonstrated vascular infiltration. The adjacent vessels were displaced in 1(11.1%) of undifferentiated embryonal carcinoma of the liver. The rest of the hepatic lesions had no effects on the adjacent vessels. No case of metastases, or regional or distant lymphadenopathy was seen.

### Rhabdomyosarcoma

Most of the cases were prostatic in origin, 2 (50%), 1 (25%) was intrapelvic with no clear organ of origin, with 1(25%) was of urinary bladder origin.

**Figures 3 a, b:**
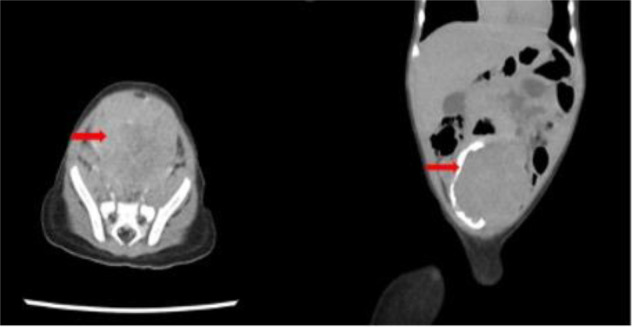
Contrast enhanced CT scan of the abdomen and pelvis of a 1-year-old female child , demonstrating a lobulated polypoid urinary bladder mass (arrows) which is outlined by contrast within the urinary bladder in b. This was confirmed to be a spindle cell sarcoma on histology

Three (75%) of the cases measured between 6 to 10cm at presentation, with 1(25%) measuring between 0 and 2cm. Three (75 %) had a heterogenous contrast-enhancing pattern on CT, and 1(25%) demonstrated a homogeneous contrast pattern. One (20%) case also showed punctate calcifications. No significant effects on the adjacent vascularity were seen in most cases (Figure 3). No distant metastases were noted in all cases.

### Germ Cell tumours

Three cases of germ cell tumours were diagnosed, out of which 2 (66.7) were malignant. These were yolk sac tumours of hepatic origin 1(33.3) and retroperitoneal gonadal dysgerminoma (33.3%). The third case was a benign mature teratoma (33.3%). At presentation, 2 (66.7%) of these tumours measured between 6 to 10cm, with 1 (33.3%) measuring 3 to 5cm. Additionally, 2 (66.7%) cases had heterogeneous enhancement on CT with no discernable calcific or fat density areas, and one (33.3%) showed homogenous enhancement. No evidence of vascular involvement, regional lymph nodes, or distant metastasis was seen.

### CT Findings and histopathological correlation

There was an overall (79.2 %) CT and histopathological concordance with a (100%) concordant in the CT pathological correlation in the case of lymphoma and germ cell tumours, and (80%) concordance for rhabdomyosarcomas, (78%) for hepatoblastoma, (50%) for neuroblastomas and (58%) for nephroblastomas. Three cases, however, had no histological diagnosis (see Table 3).

**Table 3:** Correlation between CT and histological diagnosis

	CT DIAGNOSIS	PATHOLOGICAL/ RADIOLOGICAL CORRELATION		

TUMOURS	FREQUENCY n (%)	CONCORDANT	DISCORDANT	DIAGNOSTIC ACCURACY (%)
Hepatoblastoma	8(14.3)	6	2	75
Leukaemia	3(5.4)	2	1	66.7
Lymphoma	9(16.1)	9	0	100
Malignant germ cell tumour	2(3.6)	2	0	100
Mature germ cell tumour	1(1.8)	1	0	100
Neuroblastoma	18(32.1)	10	8	50
Renal cell carcinoma	1(1.8)	0	1	0
Rhabdomyosarcoma	5(8.9)	4	1	80
Wilms	17(30.4)	10	7	58.8
Undifferentiated sarcoma of the liver	0(0)	0	1	0
Hepatocellular carcinoma	0(0)	0	1	0

## Discussion

Imaging plays a major role in the early diagnosis of tumours. By suggesting the appropriate imaging modality and providing an accurate diagnosis of early disease, the problem of late presentation of disease can be curtailed. Defining the continuum of imaging findings of these tumours in our setting will better familiarize radiologists and clinicians with the characteristics and demographics, which will ultimately help improve our diagnostic acumen and the standard of care.

This study population had slightly more males than females in a ratio of 1.1:1. In a similar study by Adesiyun et al. 8 in Nigeria, a higher male to female ratio of 1.3 :1 was probably explained by their similar demographics with their higher sample size contributing to the higher ratios. Similarly, a larger study by Choo et al., 9 suggested a higher male-to-female preponderance.

The age distribution is an important element in arriving at a differential diagnosis in this cohort of patients.^8^ In the study by Choo et al., 9 29.8% of cases were under one year, with almost 70 % being diagnosed before four years. This mirrored our finding, where most cases (66.1%) were five years or below at diagnosis. This may suggest that other factors are likely at play beyond our genetic differences.

Being conversant with the locally prevalent radiological picture is a key advantage that can narrow the diagnosis clinically, even before a histological diagnosis is made. The commonest tumours diagnosed in this study were nephroblastoma, followed by neuroblastoma, lymphoma, and hepatoblastoma. These findings were similar to that documented in a study by Zariefar et al, 10 which also found Wilms tumour to be the most common, followed by Neuroblastoma, Non Hogkins lymphoma and hepatoblastoma.

A study by Adesiyun et al , 8 showed slightly different findings with the commonest tumours being lymphoma, which was followed by Nephroblastoma, Neuroblastoma and rhabdomyosarcoma.

Bilaterality of tumours was found in 11.8 % of cases in this study. In a study by Olukayode et al,^11^ both kidneys were involved in 25% of cases, with some global studies showing bilaterality in 5 -8 % of cases, 12 The latter findings are more in keeping the finding of the index study. All cases of bilaterally occurred in males in our study population, which was at variance with the global picture, which had an overall increased female representation. 12. This could be due to our lower study population size; however, unique genetic contributions could also be a factor, thus raising questions about the need for routine tumour genetic profiling in our population to understand our situation better. CT is associated with a good detection rate for tumour thrombus,^13^ Tumour thrombus was seen in 11.8% in our study. A study by Olukayode et al. found that both tumour thrombus and the presence of lymph node metastases were seen in 17% and 50%, respectively, while the former was close to our findings and the latter was two times higher than ours. This study was associated with subcapsular hematoma in 5.9% of cases. Subscapular hematoma is indicative of preoperative tumoral rupture, be it extra or intraperitoneal as stated by the International Society of Paediatric Oncology (SIOP) guidelines. It is an indication to upstage the tumour to reduce the risk of intraperitoneal recurrence.^14^ ^15^ All cases in this study were extraperitoneal. Since first described by Sisler and Sigler in 1989, the subscapular fluid sign has become a typical feature of Rhabdoid Tumour of the Kidney (RTK). The Agrons et al. study described the sign in 77% of RTK with a further 12% of it seen in non-RT cases consisting of Wilms tumour and Clear Cell carcinoma, among others. 16

According to a consensus report from the International Neuroblastoma Risk Group Project by Brisse et al., 17 the majority of Neuroblastic tumours, including neuroblastoma, ganglioneuroblastomas and ganglioneuroma are common in the adrenal region in 48% of cases and extra-adrenal location in 24% of cases.

On the contrary, in this study, we had more than half the cases occurring in a retroperitoneal location, while a third of the cases were thought to be of adrenal origin, with 10% of cases found within the urinary bladder. These disparities again could be attributable to our smaller population size. The commonest urinary bladder malignancy is rhabdomyosarcoma. 18. Only 10 cases of urinary bladder neuroblastoma are said to have been reported worldwide to date.

When found in the urinary bladder, these tumours are known to have favourable outcomes.^19^ CT is superior in the demonstration of intratumoral calcifications. In neuroblastomata, the presence of tumoral calcifications is a vital clue. 20 A study by Xu et al. suggests that calcifications are seen in 80-90% of CTs in abdominal neuroblastoma 21, similar to what was documented in our study; 70% of cases had tumoural calcifications.

Again, these tumours are associated with high bone and lymph node metastases at diagnosis. In a large retrospective study by Zhang et al., 65.5% of cases had bone or lymphatic metastases at presentation comparable to our study which revealed regional lymph nodal metastases in 60% of cases with slightly lower proportions showing bony metastases. Their study employed MRI and Metaio-dobenzylguanidine (MIBG) scintigraphy which may have contributed to their higher yield of metastatic bone disease compared to this study.

A study by Almeida et al, with larger population size, showed that the commonest location of lymphoma was in the small bowel with 64% , mesentery 59%, retroperitoneum 26% , with fewer percentages in the colon, pelvis, liver, kidney and other locations.^22^ On the contrary our study, revealed that majority of cases were retroperitoneal, followed by bowel involvement which much like their study was dominated by small bowel involvement. Our study also revealed a small number of primary renal involvements.

Sheth et al. in their study, described the presence of multiple non-enhancing renal nodules as one of the typical findings for renal lymphoma indicative of lymphomatous deposits,23 this feature was also shown in one of our leukemia patients. It is well known that pediatric Non-Hodgkin's Lymphomas are rapidly growing tumours and are usually large at diagnosis. 24 Again, with the Almeida et al. study, over 40% of their study population had tumours measuring more than 10cm in the widest dimension at presentation.^22^ This closely mirrored our findings, with over half of our study population measuring 11 to 20cm at presentation. Other studies have suggestive worsening prognosis with increasing tumour size and number of extranodal sites. 25 No tumoral calcifications seen were seen in our study. Although extremely rare, a study by Apter et al. showed that 0.84% of pre-chemotherapy cases from a large study population of 956 had lymph node calcifications, all of whom had the aggressive type of lymphoma. 26

Due to the morphologic nature of pediatric tumours, we tend to see a wider range of tumour histological types in the liver compared to the adult liver. Malignant tumours such as hepatoblastoma, hepatocellular carcinoma, fibrolamellar carcinoma, and undifferentiated embryonal sarcomata of the liver are typically encountered, and imaging plays an important role in defining their characteristics. 27 In this study, the histological findings yielded hepatoblastoma, hepatocellular carcinoma, and embryonal carcinoma of the liver.

In a clinicopathological study by Muthuvel et al.^22^ hepatoblastoma, much like this study, was the commonest hepatic tumour. As in the Muthuvel et al study, the hepatic masses found in this current study were relatively large at presentation. Additionally, they noted that their hepatoblastoma, hepatocellular carcinomas, and some epithelioid hemangioendotheliomas were associated with raised serum Alpha Fetoprotein levels (AFP). AFP levels were, however, normal for hepatocellular adenoma and embryonal sarcoma. Our study, however, did not utilize tumour markers in our assessment. A. Chung et al. radiographic pathologic study showed that over 50% of hepatoblastomas were associated with intra-tumoural calcifications, much like this study.^28^ None of the other hepatic lesions showed intralesional calcification in our study.

### Rhabdomyosarcoma

Studies by Shelmerdine et al. indicated that 40% of rhabdomyosarcomas affected the urinary bladder and prostate gland. 29 In this study, we saw that about two-thirds of the cases had bladder and prostate masses. Their study further revealed that regardless of tumour origin, 10 to 20% of patients with rhabdomyosarcoma cases showed metastases to the lungs, cortical bone, and regional lymph nodes at presentation, denoting the aggressive nature of these tumours. Despite the generally large size of the tumours at presentation, none showed distant metastases, which could have been due to our relatively smaller sample size.

Studies have shown that malignant germ cell tumours of similar radiological phenotypes may be entirely different histologically due in part to the different pathological subtypes and high incidence of mixed tumours. One of the characteristic features of mature teratoma, however, is the presence of macroscopic fat and calcifications; these are often benign. 30

In this study, both benign and malignant cases were quite large at presentation, with a third demonstrating a heterogeneously enhancing picture and none showing any demonstrable fat or calcific component. Our histological findings included retroperitoneal gonadal dysgerminoma, primary hepatic yolk sac tumour, and mature teratoma.

Primary yolk sac tumour of the liver is extremely rare and has only been documented in case reports, the first of which was published by Hart et al. in 1975. Due to its rarity, it is imperative to exclude metastatic yolk sac tumours to the liver in such scenarios.^31^

Overall, there was approximately 76.8 % concordance for the CT and histopathology diagnosis. In evaluating retroperitoneal masses with a CT scan, a study by Vikrant et al. found the diagnostic accuracy of CT scan to be 85.7 %.^6^ Factors such as the late presentation of many of our cases, evidenced by the large tumour size at presentation leading to uncertainty in the primary organ of origin, particularly in neuroblastoma and nephroblastoma cases, may have been a limiting factor. In addition, the lack of a standardized protocol for CT studies from other imaging facilities may have contributed to the relatively lower accuracy for those tumours. Another limitation was using hard copy film instead of digital copies in several cases, some of which had been subjected to varying degrees of degradation. Finally, a small percentage of cases had no histological diagnosis.

## Conclusion

Abdominal Computed tomography is extremely instrumental in diagnosing pediatric abdominal malignancies with a high radiological and histological concordance. A wide range of abdominal malignancies was diagnosed, the commonest being neuroblastoma and nephroblastoma.
